# Isolation and Characterization of Antibiotic-Resistant and Biosurfactant-Producing Hydrocarbon-Degrading Bacteria from Industrial Contaminated Soils

**DOI:** 10.3390/toxics14070583

**Published:** 2026-07-01

**Authors:** Muhammed Yunus Emre Karaman, Hatice Ogutcu, Burak Alaylar, Medine Güllüce

**Affiliations:** 1 Vocational School of Technical Sciences, Karamanoğlu Mehmetbey University, Karaman 70200, Türkiye; myekaraman@kmu.edu.tr; 2 Department of Biology, Polatlı Faculty of Science and Letters, Ankara Hacı Bayram Veli University, Ankara 06100, Türkiye; 3 Department of Field Crops, Faculty of Agriculture, Kırsehir Ahi Evran University, Kırsehir 40100, Türkiye; 4 Department of Molecular Biology and Genetics, Faculty of Arts and Sciences, Agri Ibrahim Cecen University, Agri 04100, Türkiye; balaylar@agri.edu.tr; 5 Department of Biology, Faculty of Sciences, Atatürk University, Erzurum 25240, Türkiye; gullucem@atauni.edu.tr

**Keywords:** bioremediation, polycyclic aromatic hydrocarbons (PAHs), biosurfactant production, heavy metal resistance, industrial waste

## Abstract

In the current study, soil samples were collected from regions contaminated with industrial waste containing petroleum and its derivatives, including areas around a tire factory in Kırşehir, a chrome factory in Mersin and Kazanlı, and the Karaduvar refinery regions in Mersin, Türkiye. A total of 40 bacteria were isolated from the soil samples. The isolates were identified using molecular methods as well as morphological, physiological, and biochemical tests. Based on the results of 16S rRNA sequence analysis, it was revealed that the isolates belonged to the genera *Bacillus*, *Diaphorobacter*, *Cupriavidus*, *Acinetobacter*, *Massilia*, *Staphylococcus*, and *Azospirillum*. The antibiotic and heavy metal resistance of the strains were determined. Furthermore, the Drop Collapse method was employed to evaluate the biosurfactant production abilities of the strains, confirming that certain strains possess biosurfactant-producing capabilities. Consequently, distinct bacterial species capable of degrading polyaromatic hydrocarbons, resisting antibiotics and heavy metals, and producing biosurfactants were successfully isolated from contaminated areas in the current study. It is thought that the utilization of species with these properties in bioremediation studies will contribute to the restoration of ecosystem balance.

## 1. Introduction

The rapid increase in industrial development, urbanization, and the intensive use of agrochemicals and anthropogenic activities has introduced numerous pollutants into the environment, such as pesticides, herbicides, heavy metals, and petroleum-based products [1]. Among these hazardous industrial wastes, petroleum and its derivatives cause critical damage to aquatic and terrestrial ecosystems due to high global demand. Today, a significant portion of the energy resources required for industrial and domestic needs is met by oil and petroleum products [2,3]. It is well established that petroleum and its derivatives contain various detrimental components, including n-alkanes, cycloalkanes, and PAHs [4]. Hydrocarbons derived from oil, such as diesel, kerosene, and petrol, frequently contaminate soil and surrounding ecosystems during oil drilling, fuel waste disposal, and accidental spills from tanker ships [5,6].

The interaction of petroleum residues with nature alters their original properties due to the combined effects of numerous physical and biological factors. The long-term presence of these hydrocarbon-containing derivatives leads to environmental pollution, resulting not only in the death of the affected flora and fauna but also in various genetic mutations [1,7]. Moreover, chronic exposure to excessive petroleum can lead to internal organ diseases, bone marrow damage, and a high risk of cancer due to the presence of mutagenic substances [7,8]. Consequently, there is an increasing demand for remediation methods that alleviate these problems while minimizing harm to the ecosystem. Various approaches, including chemical, physical, thermal, and biological strategies, have been developed [9,10]. However, biological applications, such as microbial bioremediation, offer more eco-friendly and cost-effective approaches that are gaining wider attention [11,12].

To date, numerous hydrocarbon-degrading bacteria have been isolated and molecularly identified from industrial wastes, as well as from soil and water exposed to oil products [5,13,14]. Molecular identifications have revealed that genera such as *Acinetobacter*, *Alcaligenes*, *Arthrobacter*, *Bacillus*, *Corynebacterium*, *Micrococcus*, *Pseudomonas*, *Methylobacterium*, and *Rhodococcus* are among the most common hydrocarbon degraders. Many of these bacteria also exhibit resistance to heavy metals and antibiotics, alongside biosurfactant-producing properties [15,16]. Furthermore, PAH contamination in industrial areas is often coupled with the presence of heavy metals. Heavy metal contamination can negatively affect the bioremediation of hydrocarbons due to the synergistic cytotoxic effects of these dual contaminants. Therefore, searching for bacterial strains that can degrade PAHs while exhibiting high resistance to heavy metals in the soil has become a critical research priority [12]. Additionally, the biological degradation capacity of microorganisms is often restricted by the hydrophobic nature of petroleum. While chemical surfactants have been developed to enhance degradation, microbial-derived biosurfactants are more effective for decontaminating oil spills due to their superior biodegradability [17].

The present study aimed to isolate and characterize native bacterial species from industrial waste-contaminated areas and petroleum and its derivatives that are capable of degrading polyaromatic hydrocarbons, resisting antibiotics and heavy metals, and producing biosurfactant, predicting the ability of strains in various environmental conditions.

## 2. Materials and Methods

### 2.1. Collection of Soil Samples

Soil samples were collected from industrial areas contaminated with petroleum and its derivatives in Türkiye. The sampling sites included a tire factory in Kırşehir, a chromium factory in Mersin, and refinery regions in the Kazanlı and Karaduvar districts of Mersin. Samples were collected aseptically, stored in sterile plastic zip-lock bags, and transported to the Microbiology Laboratory at Kırşehir Ahi Evran University. All samples were maintained at 4 °C until further analysis.

### 2.2. Crude Oil and Reference Strain

The crude oil used in this study was obtained from the Aliağa Tüpraş Petroleum Refinery (İzmir, Türkiye). *Pseudomonas aeruginosa* ATCC 27853 (Boston-USA) was utilized as the reference strain (it was used because it belongs to the genus (*Pseudomonas*) and species that degrade hydrocarbons).

### 2.3. Isolation and Screening of Hydrocarbon Degrading Bacteria

For the isolation process, a mineral salt medium (MSM) was prepared containing 1.0 g KNO_3_, 0.2 g MgSO_4_, 0.1 g NaCl, 0.1 g CaCl_2_, and 1.0 g K_2_HPO_4_ per liter. The chemicals were purchased from Merck (Darmstadt, Germany). To this, 1% crude oil/Triton-X-100 (1:1) was added as the carbon source. Ten grams of soil sample were inoculated into 100 mL of this medium and incubated in a shaker incubator (MAXQ 4450) at 28 °C and 180 rpm for three days. Following this, 10 mL of the culture was transferred into fresh medium for a second round of enrichment; this process was repeated a total of three times. After the final incubation, 0.1 mL of the culture was spread onto Nutrient Agar (NA) plates. All experiments were repeated three times. Morphologically distinct colonies were selected using a binocular microscope (Novex P-20, Euromex Microscopen bv, Arnhem, The Netherlands) and isolated for further characterization [18,19].

### 2.4. Morphological and Biochemical Characterization

The isolated hydrocarbon-degrading strains were subcultured on NA medium. Morphological characteristics, including colony shape and color, were recorded. Then, Gram staining, 3% KOH, motility, catalase, and oxidase tests were exploited for assessment of morphological, physiological, and biochemical characterizations of bacterial strains by utilizing conventional methods according to the Harley and Prescott (2002) method [20].

### 2.5. Determination of Antibiotic Resistance

Antibiotic susceptibility testing was conducted on Mueller–Hinton Agar (MHA) using the disc diffusion method. The following ten antibiotics were utilized: Ampicillin (AM, 10 μg), Nalidixic acid (NA, 30 μg), Chloramphenicol (C, 30 μg), Tetracycline (TE, 30 μg), Nitrofurantoin (F/M, 300 μg), Gentamicin (GM, 10 μg), Imipenem (IPM, 10 μg), Ciprofloxacin (CIP, 5 μg), Cefotaxime (CTX, 30 μg), and Erythromycin (E, 15 μg). *P. aeruginosa* ATCC 27853 served as the reference strain to ensure antibacterial efficacy [21]. Isolates were activated in nutrient broth, adjusted to a 0.5 McFarland turbidity standard, and inoculated onto MHA plates using the spread-plating technique. Antibiotic discs were placed at specific intervals. Following incubation at 28 °C for 24 h, the diameters of the inhibition zones were measured and recorded in millimeters [22].

### 2.6. Determination of Heavy Metal Resistance Levels

Metals such as cobalt (Co), zinc (Zn), manganese (Mn) and mercury (Hg) are among the most well-known heavy metals for bioremediation studies [23]. In this regard, heavy metal resistance was evaluated using the well-diffusion method [23]. Based on preliminary experiments, concentrations were set at 5, 10, 15, and 20 mM to identify the most resistant strains. Aqueous solutions of CoCl_2_·6H_2_O, ZnSO_4_·7H_2_O, MnCl_2_·2H_2_O, and HgCl_2_ were sterilized using 0.2 μm PTFE filters before being added to the media. *P. aeruginosa* ATCC 27853 was used as the control. Strains exhibiting growth at higher metal concentrations than the control were considered resistant.

### 2.7. Cultivation for Biosurfactant Production

To induce biosurfactant production, stock cultures were first activated in Tryptic Soy Broth (TSB). The activated cultures were then inoculated into Mineral Salt Medium (MSM) at a 1/20 (*v*/*v*) ratio under sterile conditions. Incubation was performed at 35 °C for 10 days in a shaking incubator (Thermo MAXQ 4450, Thermo Fisher Scientific, Marietta, OH, USA) at 150 rpm [24].

### 2.8. Detection of Biosurfactant Activity

Biosurfactant production was detected using the “Drop Collapse” method. Cultures were centrifuged at 10,000 rpm for 20 min (Micro CL 17), and the supernatants were filtered through 0.2 μm PTFE membranes. For the assay, 96-well microwell plates were coated with 7 μL of mineral oil and equilibrated at room temperature for 24 h. Subsequently, 25 μL of the filtrate was dropped onto the oil surface at a 45° angle. Sterile water and uninoculated medium served as negative controls. Biosurfactant activity was confirmed by observing the collapse or spreading of the droplets compared to the stable droplets of the controls [24].

### 2.9. Molecular Identification via 16S rRNA

Molecular identification of selected bacterial strains was realized by using 16S rRNA gene sequencing. Genomic DNA extraction was performed according to the following method, and the 16S rDNA gene region was amplified via Polymerase Chain Reaction (PCR) using the universal primers 27F (5′-AGAGTTTGATCMTGGCTCAG-3′) and 1492R (5′-GGTTACCTTGTTACGACTT-3′). The 30 μL reaction mixture contained 10× PCR buffer, 10 mM dNTPs, 1.2 μL DMSO, 50 μM MgCl_2_, 0.3 U/mL Taq DNA polymerase, and 100 ng/μL template DNA. PCR amplification was performed with an initial denaturation at 95 °C for 2 min, followed by 36 cycles of 94 °C for 1 min, 54 °C for 1 min, and 72 °C for 2 min, with a final extension at 72 °C for 5 min. The resulting PCR products were analyzed using 1% agarose gel electrophoresis, stained with ethidium bromide, and visualized under a gel documentation system (DNR Bio-Imaging Systems).

## 3. Results

### 3.1. Morphological and Biochemical Characterization

In the present study, a total of 40 bacterial strains were isolated from the specified contaminated sites. Morphological analysis revealed that 25 isolates (62.5%) exhibited smooth (S-type) colonies, 12 (30%) showed rough (R-type) colonies, 2 (5%) were mucoid (M-type), and 1 (2.5%) was characterized as L-type. Gram staining results indicated that 17 isolates were G (+), while 23 were G(*−*). These results were further confirmed by the 3% KOH test, which yielded 23 positive and 17 negative results, consistent with the Gram reactions. Regarding motility, 24 isolates were found to be motile, while 16 were non-motile.

Biochemical testing showed that 38 isolates were catalase-positive, forming characteristic gas bubbles upon the addition of hydrogen peroxide, whereas only 2 isolates were catalase-negative. In the oxidase test, 25 isolates (62.5%) tested positive by oxidizing the p-aminodimethylaniline reagent to a purple-blue color, while 15 isolates were oxidase-negative. The morphological and biochemical properties of the strains with the highest hydrocarbon-degrading potential are summarized in Table 1. (Note: *P. aeruginosa* ATCC 27853 was excluded from these specific counts.)

### 3.2. Antibiotic Resistance Profiles

The resistance rates among the 20 selected isolates were as follows: Tetracycline (75%), Nitrofurantoin (65%), Ampicillin (60%), Nalidixic Acid (60%), Ciprofloxacin (55%), Erythromycin (50%), Chloramphenicol (45%), Cefotaxime (40%), Gentamicin (25%), and Imipenem (15%).

**Figure 1:**
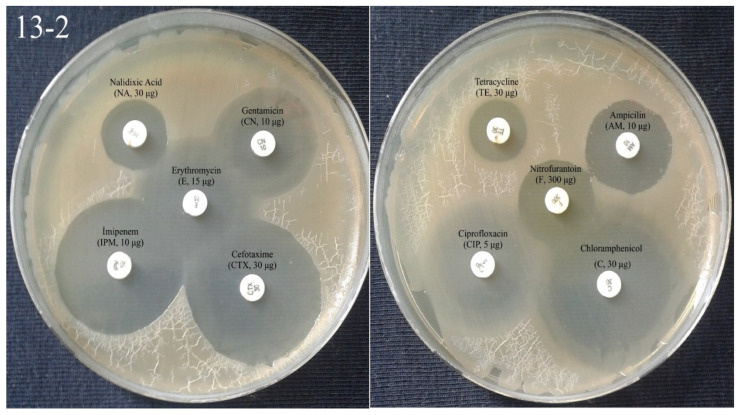
Antibiotics resistance results of YH13-2.

Tetracycline was identified as the antibiotic with the highest resistance rate (75%), whereas Imipenem proved to be the most effective, with only 15% resistance. Notably, isolate YH 13-1 exhibited multidrug resistance against all tested antibiotics. Conversely, isolates YH 13-4 and YH 16-1 were found to be susceptible to all antibiotics (Table 2 and Figure 1).

### 3.3. Determining Heavy Metal Resistance Levels of Bacterial Strains

**Figure 2:**
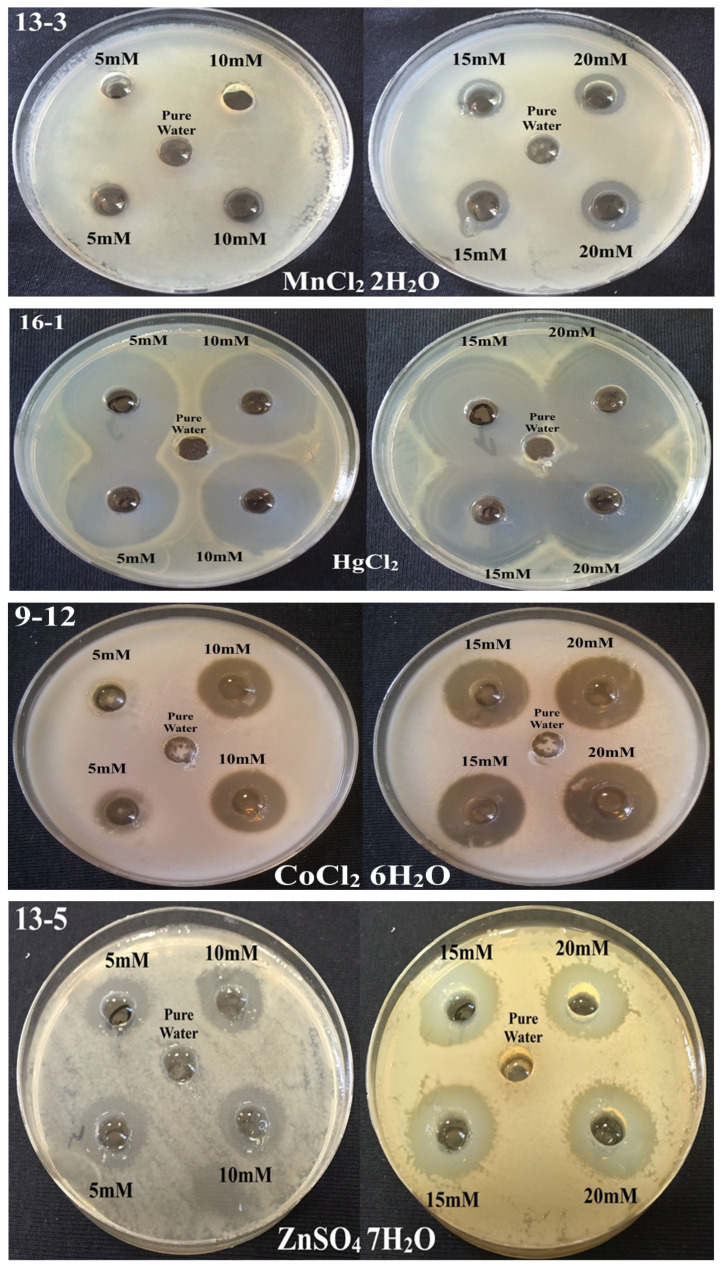
Heavy metal resistance results of some isolates.

Heavy metal resistance was evaluated using *P. aeruginosa* ATCC 27853 as a control strain (Table 3). For cobalt, 25% of the isolates were resistant to 5 mM, 5% to 10 mM, and 10% to 20 mM, while 60% were sensitive. Regarding zinc, 10% were resistant to 5 mM and 10 mM, and 15% to 20 mM; however, 65% of the total isolates showed resistance to cobalt at various levels. Manganese resistance was significantly higher, with 40% of the isolates tolerating concentrations up to 20 mM. In contrast, resistance to mercury was minimal; only isolate YH 10-12 exhibited resistance at 5 mM, while all other isolates were sensitive across all tested concentrations (Figure 2). Isolate YH 13-1 was found to be resistant to all antibiotics, as well as heavy metals except HgCl_2_. Also, similar results were found in the other bacterial strains (Table 2 and Table 3).

### 3.4. Biosurfactant Production

Based on the results, 15 out of 20 isolates (75%) demonstrated biosurfactant production, while 5 isolates (YH 9-4, YH 9-6, YH 13-3, YH 13-4, and YH 15-2) did not exhibit this ability. The YH 13-1 isolate, which tested positive for biosurfactant production, was found to be resistant to both antibiotics and heavy metals (except HgCl_2_). Detailed results are presented in Table 4.

### 3.5. Molecular Identification

**Figure 3:**
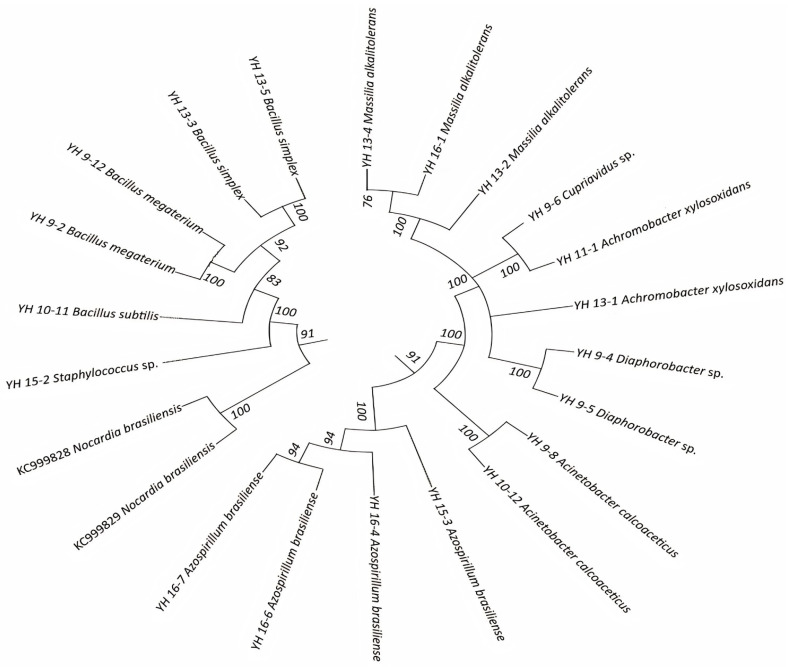
Neighbor-joining phylogenetic tree based on 16S rRNA gene sequences of the strains.

The 16S rDNA sequences of 20 isolates were determined and compared with the GenBank database using BLAST analysis. Identification results, including base pair numbers and similarity percentages, are provided in Table 5. A neighbor-joining phylogenetic tree was constructed using MEGA 12 software [21] to illustrate the evolutionary relationships of the strains (Figure 3).

## 4. Discussion

Since the 19th century, industrially extracted crude oil often referred to as “black gold” has become the world’s most essential energy source, underpinning the economies of many nations [8,25]. However, the environmental cost of this dependency is significant. Anthropogenic activities release approximately 8 million tons of crude oil into the environment annually, leading to severe soil and water contamination [25]. Conventional remediation methods, such as adsorption and ozone catalytic oxidation, provide rapid solutions but are often limited by high costs and the risk of secondary contamination [10]. Consequently, biotechnological approaches like bioremediation have become a cornerstone for the sustainable elimination of petroleum hydrocarbons, utilizing native microorganisms to convert toxins into non-toxic metabolites [12].

In the current study, 40 bacterial strains were isolated from industrial sites in Türkiye (Kırşehir and Mersin), with 20 isolates identified as high-potential petroleum degraders. These isolates belong to the genera *Bacillus*, *Diaphorobacter*, *Cupriavidus*, *Acinetobacter*, *Achromobacter*, *Massilia*, *Staphylococcus*, and *Azospirillum*. Our findings are strongly supported by previous literature that identifies these genera as key players in hydrocarbon degradation and environmental resilience.

For instance, Abbaszade et al. (2020) utilized *Cupriavidus campinensis* as a model organism to demonstrate simultaneous heavy metal and antibiotic resistance alongside aromatic compound degradation [26]. Our isolation of *Cupriavidus* sp. from refinery areas mirrors these results, confirming the genus’s robust biotechnological potential. Similarly, Al Disi et al. (2022) isolated *Bacillus* and *Pseudomonas* strains from oily soils in Qatar, which achieved 70–80% heavy metal removal and 73% diesel degradation. This aligns with our observation of *Bacillus megaterium* and *Bacillus subtilis* exhibiting high survival rates in multi-polluted soils [12].

The identification of *Diaphorobacter* sp. in our study is consistent with reports by Wen et al. (2022), who found that *Diaphorobacter* sp. strain MNS-0 could impressively degrade phenanthrene while maintaining resistance to a wide spectrum of antibiotics, including ampicillin and chloramphenicol [27]. This parallels our results where our *Diaphorobacter* isolates showed multidrug resistance. It is well known that heavy metal and antibiotic resistance genes often co-localize on plasmids, facilitating horizontal gene transfer and activating survival pathways in multi-pollutant environments. This co-resistance provides dual adaptive advantages that sustain bacterial viability in contaminated sites. Consequently, these plasmids drive efficient bioremediation through the simultaneous detoxification and catabolism of organic hydrocarbons and inorganic co-contaminants [28].

Furthermore, the isolation of *Achromobacter xylosoxidans* in our study supports the work of Kopella et al. (2023), who identified this species as a potent biosurfactant producer capable of emulsifying lubricant oil-contaminated soils [29].

Our findings regarding *Massilia alkalitolerans* and *Azospirillum brasilense* also find support in the recent literature. Li et al. (2021) successfully utilized *Massilia* sp. for phenanthrene degradation [13], while Nanekar and Kokitkar (2023) identified *Azospirillum* sp. as a key degrader in petroleum-contaminated regions in India [9]. Moreover, the biosurfactant production capabilities of our *B. megaterium* and *Acinetobacter calcoaceticus* isolates are corroborated by Shokranian et al. (2025) and Hoa Kieu et al. (2025), who highlighted these species as promising strains for the biodegradation of complex hydrocarbons [16,17].

The diversity of genera found in our study extending to *Staphylococcus* mirrors the findings of Omrani et al. (2025), who identified similar hydrocarbon-utilizing strains in diverse environments in Tunisia. Collectively, these comparisons validate that our native isolates possess the necessary metabolic machinery for effective bioremediation. As the global shift toward green technology intensifies, the use of these multi-functional bacterial strains offers a reliable, permanent, and economically viable technique for cleaning polluted territories [30]. Especially, *P. aeruginosa* ATCC 27853 strains possess the ability to degrade and process petroleum-based compounds. They produce specific enzymes that pseudo-solubilize hydrocarbons, making it easier for them to detach from soil particles. This ability gives these microorganisms great potential for the bioremediation of a crude oil-polluted environment [31].

Overall, while limited studies have focused on functional bacterial communities and their microbial diversity during the bioremediation process, our findings confirm our hypothesis that native functional bacterial populations play pivotal roles in the bioremediation of PAH-contaminated soil. Hence, this study successfully identified diverse bacterial strains with high potential to degrade PAHs in contaminated regions, offering a more extensive perspective on the bioremediation of soils industrially contaminated with PAHs and their derivatives. Therefore, our study is unique in that it demonstrates in detail the specific bacterial strains that can be used in remediating areas contaminated with industrial wastes containing petroleum and petroleum derivatives from the chromium factory and the Kazanlı and Karaduvar refinery regions in Mersin, Türkiye.

## 5. Conclusions

The present study highlights microbial bioremediation as an environmentally friendly and cost-effective strategy to mitigate the impact of petroleum-derived pollutants on human life and the environment. Our results demonstrate that the isolated bacterial strains, characterized by their high hydrocarbon degradation potential, biosurfactant production, and resistance to both heavy metals and antibiotics, offer a viable solution for the remediation of industrial waste-contaminated sites. Among these strains, YH 13-1 was one of the best hydrocarbon-degrading bacteria and found to be resistant to all antibiotics, as well as heavy metals except HgCl_2_. Also, similar results were found in the other bacterial strains. These native isolates represent promising candidates for restoring ecosystem balance in regions heavily impacted by petroleum pollution.

## Figures and Tables

**Table 1:** Morphological, physiological and biochemical characterization results for bacterial isolates.

Bacterial Code	Cell Morphology	Gram Property	Motility	KOH (%3)	Catalase Activity	Oxidase Activity
**YH 9-2**	Bacilli	+	+	−	+	−
**YH 9-4**	Bacilli	−	−	+	+	+
**YH 9-5**	Bacilli	−	−	+	+	+
**YH 9-6**	Bacilli	−	−	+	+	+
**YH 9-8**	Coccobacillus	−	−	+	+	−
**YH 9-12**	Bacilli	+	+	−	+	+
**YH 10-11**	Bacilli	+	+	−	+	+
**YH 10-12**	Coccobacillus	−	−	+	+	−
**YH 11-1**	Bacilli	−	+	+	+	+
**YH 13-1**	Bacilli	−	+	+	+	+
**YH 13-2**	Bacilli	−	+	+	+	+
**YH 13-3**	Bacilli	+	−	−	+	−
**YH 13-4**	Bacilli	+	−	−	+	−
**YH 13-5**	Bacilli	+	+	−	+	−
**YH 15-2**	Bacilli	+	−	−	+	−
**YH 15-3**	Bacilli	−	−	+	+	+
**YH 16-1**	Bacilli	−	−	+	+	+
**YH 16-4**	Bacilli	−	−	+	−	−
**YH 16-6**	Bacilli	−	+	+	+	+
**YH 16-7**	Bacilli	−	+	+	+	+

“+” as positive, “−” as negative.

**Table 2:** Antibiotic resistance of bacterial strains.

Bacterial Code	Nalidixic Acid (NA, 30 μg)	Imipenem (IPM, 10 μg)	Cefotaxime (CTX, 30 μg)	Gentamicin (GM, 10 μg)	Erythromycin (E, 15 μg)
**YH 9-2**	R	S	S	S	S
**YH 9-4**	S	S	S	S	R
**YH 9-5**	S	S	R	R	R
**YH 9-6**	S	R	S	R	R
**YH 9-8**	R	S	R	S	R
**YH 9-12**	R	S	S	S	S
**YH 10-11**	R	S	S	S	S
**YH 10-12**	R	S	R	R	R
**YH 11-1**	R	S	S	R	S
**YH 13-1**	R	R	R	R	R
**YH 13-2**	S	S	S	S	S
**YH 13-3**	R	S	R	S	S
**YH 13-4**	S	S	S	S	S
**YH 13-5**	R	S	S	S	S
**YH 15-2**	R	S	S	S	R
**YH 15-3**	R	S	R	S	S
**YH 16-1**	S	S	S	S	S
**YH 16-4**	S	S	S	S	R
**YH 16-6**	S	S	S	S	R
**YH 16-7**	R	S	R	S	R
***P. aeruginosa***					
**ATCC 27853**	R	R	R	S	R
**Bacterial Code**	**Tetracycline (TE, 30 μg)**	**Ciprofloxacin (CIP, 5 μg)**	**Chloramphenicol (C, 30 μg)**	**Ampicilli** **n** **(AM, 10 μg)**	**Nitrofurantoin (F/M, 300 μg)**
**YH 9-2**	R	S	R	R	R
**YH 9-4**	R	S	S	R	R
**YH 9-5**	R	S	S	R	R
**YH 9-6**	R	R	S	R	R
**YH 9-8**	R	R	R	R	R
**YH 9-12**	R	S	R	S	R
**YH 10-11**	R	R	S	R	R
**YH 10-12**	R	R	R	R	R
**YH 11-1**	R	R	S	S	R
**YH 13-1**	R	R	R	R	R
**YH 13-2**	R	S	S	S	S
**YH 13-3**	R	R	R	R	R
**YH 13-4**	S	S	S	S	S
**YH 13-5**	S	R	S	S	S
**YH 15-2**	R	R	S	R	R
**YH 15-3**	R	R	S	R	R
**YH 16-1**	S	S	S	S	S
**YH 16-4**	S	S	S	R	S
**YH 16-6**	R	S	R	S	S
**YH 16-7**	S	S	R	R	S
***P. aeruginosa***					
**ATCC 27853**	R	R	R	S	R

**Table 3:** Heavy metal resistance of bacterial strains.

Bacterial Isolates	Concentration (mM)	CoCl_2_ 6H_2_O	ZnSO_4_ 7H_2_O	MnCl_2_ 2H_2_O	HgCl_2_
**YH 9-2**	5	S	S	R	S
	10	S	S	R	S
	15	S	S	R	S
	20	S	S	R	S
**YH 9-4**	5	R	S	R	S
	10	S	S	R	S
	15	S	S	S	S
	20	S	S	S	S
**YH 9-5**	5	R	S	R	S
	10	S	S	S	S
	15	S	S	R	S
	20	S	S	S	S
**YH 9-6**	5	S	R	R	S
	10	S	R	R	S
	15	S	R	R	S
	20	S	R	R	S
**YH 9-8**	5	S	R	R	S
	10	S	S	R	S
	15	S	S	S	S
	20	S	S	S	S
**YH 9-12**	5	S	S	R	S
	10	S	S	R	S
	15	S	S	R	S
	20	S	S	R	S
**YH 10-11**	5	R	S	S	S
	10	S	S	S	S
	15	S	S	S	S
	20	S	S	S	S
**YH 10-12**	5	S	R	R	R
	10	S	R	R	S
	15	S	R	S	S
	20	S	R	S	S
**YH 11-1**	5	S	R	R	S
	10	S	S	R	S
	15	S	S	R	S
	20	S	S	R	S
**YH 13-1**	5	R	R	R	S
	10	R	R	R	S
	15	R	R	R	S
	20	R	R	R	S
**YH 13-2**	5	R	R	S	S
	10	R	R	S	S
	15	S	S	S	S
	20	S	S	S	S
**YH 13-3**	5	R	S	R	S
	10	S	S	R	S
	15	S	S	R	S
	20	S	S	R	S
**YH 13-4**	5	R	S	S	S
	10	R	S	S	S
	15	R	S	S	S
	20	R	S	S	S
**YH 13-5**	5	R	S	R	S
	10	S	S	R	S
	15	S	S	S	S
	20	S	S	S	S
**YH 15-2**	5	S	S	R	S
	10	S	S	S	S
	15	S	S	S	S
	20	S	S	S	S
**YH 15-3**	5	S	S	R	S
	10	S	S	R	S
	15	S	S	R	S
	20	S	S	S	S
**YH 16-1**	5	S	R	R	S
	10	S	R	S	S
	15	S	S	S	S
	20	S	S	S	S
**YH 16-4**	5	S	S	R	S
	10	S	S	R	S
	15	S	S	R	S
	20	S	S	R	S
**YH 16-6**	5	S	S	R	S
	10	S	S	R	S
	15	S	S	R	S
	20	S	S	R	S
**YH 16-7**	5	S	S	R	S
	10	S	S	R	S
	15	S	S	R	S
	20	S	S	R	S

**Table 4:** Biosurfactant production results of isolates.

Bacterial Code	Biosurfactant Production
YH 9-2	+
YH 9-4	−
YH 9-5	+
YH 9-6	−
YH 9-8	+
YH 9-12	+
YH 10-11	+
YH 10-12	+
YH 11-1	+
YH 13-1	+
YH 13-2	+
YH 13-3	−
YH 13-4	−
YH 13-5	+
YH 15-2	−
YH 15-3	+
YH 16-1	+
YH 16-4	+
YH 16-6	+
YH 16-7	+
*Pseudomonas aeruginosa* ATCC 27853	+

“+” as positive, “−” as negative.

**Table 5:** GenBank accession numbers and information of the bacterial strains.

Bacterial Code	Base Number	Name	Simillarity (%)	Genbank Number
**YH 9-2**	1418	*Bacillus megaterium*	100	KY010267
**YH 9-4**	1344	*Diaphorobacter* sp.	100	KY010268
**YH 9-5**	1346	*Diaphorobacter* sp.	100	KY010269
**YH 9-6**	1392	*Cupriavidus* sp.	99	KY010270
**YH 9-8**	1396	*Acinetobacter calcoaceticus*	99	KY010271
**YH 9-12**	1415	*Bacillus megaterium*	100	KY010272
**YH 10-11**	1429	*Bacillus subtilis*	100	KY010273
**YH 10-12**	1379	*Acinetobacter calcoaceticus*	100	KY010274
**YH 11-1**	1373	*Cupriavidus* sp.	99	KY010275
**YH 13-1**	1380	*Achromobacter xylosoxidans*	100	KY010276
**YH 13-2**	1382	*Massilia alkalitolerans*	99	KY010277
**YH 13-3**	1399	*Bacillus simplex*	100	KY010278
**YH 13-4**	1364	*Massilia alkalitolerans*	99	KY010279
**YH 13-5**	1421	*Bacillus simplex*	99	KY010280
**YH 15-2**	1422	*Staphylococcus* sp.	100	KY010281
**YH 15-3**	1303	*Azospirillum brasilense*	99	KY010282
**YH 16-1**	1352	*Massilia alkalitolerans*	99	KY010283
**YH 16-4**	1015	*Azospirillum brasilense*	100	KY010284
**YH 16-6**	1017	*Azospirillum brasilense*	99	KY010285
**YH 16-7**	1329	*Azospirillum brasilense*	98	KY010286

## Data Availability

The original contributions presented in this research are included in the article. Further inquiries can be directed to the corresponding authors.

## Abbreviations

The following abbreviations are used in this manuscript:

PAHs	Polycyclic Aromatic Hydrocarbons

## References

[1] Ayilara M.S., Babalola O.O. (2023). Bioremediation of environmental wastes: The role of microorganisms. Front. Agron..

[2] Benmessaoud S., Anissi J., Kara M., Assouguem A., Al-Huqail A.A., Germoush M.O., Ullah R., Ercisli S., Bahhou J. (2022). Isolation and characterization of three new crude oil degrading yeast strains, *Candida parapsilosis* SK1, *Rhodotorula mucilaginosa* SK2 and SK3. Sustainability.

[3] Mesbah A., Chaib N., Boudjellab Z.E., Ghannam M., Djazi F. (2026). Halotolerant indigenous bacterial–fungal consortia for biodegradation of petroleum hydrocarbons from Algerian refinery sludge: An integrated molecular and biodegradation approach. Biodegradation.

[4] Okoye A.U., Selvarajan R., Chikere C.B., Okpokwasili G.C., Mearns K. (2024). Characterization and identification of long-chain hydrocarbon-degrading bacterial communities in long-term chronically polluted soil in Ogoniland: An integrated approach using culture-dependent and independent methods. Environ. Sci. Pollut. Res..

[5] Ogutcu H., Kantar F., Alaylar B., Numanoglu Y., Gulluce M. (2022). Isolation and characterization of hydrocarbon and petroleum degrading bacteria from polluted soil with petroleum and derivatives by MALDI-TOF MS method. Geomicrobiol. J..

[6] Ravi A., Ravuri M., Krishan R., Narenkumar J., Anu K., Alsalhi M.S., Devanesan S., Kamala-Kannan S., Rajasekar A. (2022). Characterization of petroleum degrading bacteria and its optimization conditions on effective utilization of petroleum hydrocarbons. Microbiol. Res..

[7] Tahir U., Zameer M., Frrukh S.Y., Ghauri B.A., Javed M.A., Ali Q., Ali D. (2024). Isolation, and characterization of petroleum oil-degrading bacteria from petroleum contaminated soil. Geomicrobiol. J..

[8] Wang R., Wu B., Zheng J., Chen H., Rao P., Yan L., Chai F. (2020). Biodegradation of total petroleum hydrocarbons in soil: Isolation and characterization of bacterial strains from oil contaminated soil. Appl. Sci..

[9] Nanekar R.D., Kokitkar S.S. (2023). Isolation, genetic identification and optimization of hydrocarbon degrading bacteria from petroleum contaminated soil in Raigad region. Eco. Env. Cons..

[10] Uz Zaman S.A., Bhrdwaj A., Nayarisseri A., Khazanehdari K.A., Bhuyan R. (2025). Isolation and characterization of novel hydrocarbon-degrading bacteria from oil polluted soil near Nacharam, Hyderabad, India. Sci. Rep..

[11] Sezen S., Güllüce M., Karadayi M., Alaylar B. (2020). First report of fungal strains from Afşin–Elbistan mine for microbial lignite process. Geomicrobiol. J..

[12] Al Disi Z., Al-Ghouti M., Zouari N. (2022). Investigating the simultaneous removal of hydrocarbons and heavy metals by highly adapted *Bacillus* and *Pseudomonas* strains. Environ. Technol. Innov..

[13] Li Q., Li J., Jiang L., Sun Y., Luo C., Zhang G. (2021). Diversity and structure of phenanthrene degrading bacterial communities associated with fungal bioremediation in petroleum contaminated soil. J. Hazard. Mater..

[14] Lucchina P.G., Catania V., Trapani D.D., Petta E.M., Calabrisotto L.S., Vinti G., Quatrini P., Viviani G. (2026). Sequential biostimulation and bioaugmentation treatments of a diesel-contaminated soil: Effect on hydrocarbon degradation and soil bacterial communities. Appl. Soil Ecol..

[15] Kainat K., Rehman A., Hussain M., Qasim M., Khattak B., Naz I. (2025). Bacterial-augmented phytoremediation of petroleum hydrocarbons using *Triticum aestivum* and *Zea mays*. Int. J. Phytoremediat..

[16] Shokranian S., Yousefzadi M., Biuki N.A., Yaghoubi-Avini M., Akerdi M.T. (2025). Isolation, characterization, and bioremediation potential of oil-degrading bacteria from contaminated soils in Shiraz and Bandar Abbas, Iran. Sci. Rep..

[17] Hoa Kieu T.Q., Nguyen T.Y., Nguyen V.T., Nguyen T.N.Q. (2023). Biosurfactant production by a crude oil-utilizing bacterium towards petroleum hydrocarbon biodegradation applications. Vietnam J. Chem..

[18] Rojas-Avelizapa N.G., Rodriguez-Vazquez R., Enriquez-Villanueva F., Martinez-Cruz J., Poggi-Varaldo H.M. (1999). Transformer oil degradation by an indigenous microflora isolated from a contaminated soil. Resour. Conserv. Recycl..

[19] Eraydın-Erdoğan E. (2010). Studies a Bioremediation of Crude Oil Polluted Soil in Laboratory Conditions. Ph.D. Thesis.

[20] Harley J.P., Prescott L.M. (2002). Laboratory Exercises in Microbiology.

[21] (1997). Performance Standards for Antimicrobial Disc Susceptibility Tests, 6th ed.

[22] Akkan T. (2009). Determination of Antibiotic and Heavy Metal Resistance and Plasmid Profile of Bacteria in İskenderun Bay. Master’s Thesis.

[23] Nithya C., Gnanalakshmi B., Pandian S.K. (2011). Assessment and characterization of heavy metal resistance in palk bay sediment bacteria. Mar. Environ. Res..

[24] Yalçın E. (2008). Biosurfactant Production and Investigation of Hydrocarbon Degredation with Microorganisms Isolated from Refinery Wastewaters. Ph.D. Thesis.

[25] Tang F., Zhang H., Hao C., Wang Y., Li Q., Zhao C., Gu Y., Wang J. (2023). New insights of crude oil biodegradation construction by microbial consortium B10: Responded substrates, genomics, biodegradation mechanism and pathways. J. Chem. Eng..

[26] Abbaszade G., Szabó A., Vajna B., Farkas R., Szabó C., Tóth E. (2020). Whole genome sequence analysis of *Cupriavidus campinensis* S14E4C, a heavy metal resistant bacterium. Mol. Biol. Rep..

[27] Wen L., Huang Y., Wang W., Zhang L., Xu J., Li Z., Xu P., Tang H. (2022). A novel *Diaphorobacter* sp. strain isolated from saponification wastewater shows highly efficient phenanthrene degradation. Environ. Res..

[28] Limaa T., Batmunkh M., Dulguun M., Dorjsuren B., Turmunkh T. (2025). Bacterial heavy metal resistance in contaminated soil. J. Microbiol. Biotechnol..

[29] Kopalle P., Pothana S.A. (2023). Biodegradation of waste lubricant oil by a novel ısolated biosurfactant producer- *Achromobacter xylosoxidans* PSA5. Geomicrobiol. J..

[30] Omrani R., Spini G., Puglisi E., Saidane D. (2025). Isolation and characterization of hydrocarbon- degrading bacteria from polluted terrestrial and aquatic environments in Tunisia. Soil Sediment Contam..

[31] Baig Z.T., Abbasi S.A., Memon A.G., Naz A., Soomro A.F. (2022). Assessment of degradation potentialof *Pseudomonas* species in bioremediatingsoils contaminated with petroleumhydrocarbons. J. Chem. Technol. Biotechnol..

